# Supplementary material for: Association between Urban Greenness and Depressive Symptoms: Evaluation of Greenness Using Various Indicators
Source: Int J Environ Res Public Health. 2019 Jan 9;16(2):173. doi: 10.3390/ijerph16020173 (PMC6352234; doi:10.3390/ijerph16020173)
Supplement: Supplementary file 1 [file ijerph-16-00173-s001.pdf]

Supplementary Information

Association between Urban Greenness and Depressive Symptoms: Evaluation of Greenness Using Various Indicators

Hyeonjin Song [1], Kevin James Lane [2], Honghyok Kim [3], Hyomi Kim [3], Garam Byun [3], Minh Le [3], Yongsoo Choi [3], Chanryul Park [1] and Jong-Tae Lee [3,4,*]

[1] Urban Forests Research Center, National Institute of Forest Science, Seoul 02455, Korea; etaoin0810@naver.com (H.S.); maeulsoop@korea.kr (C.P.)
[2] School of Public Health, Boston University, Boston, MA 02118, USA; klane@bu.edu
[3] BK21PLUS Program in 'Embodiment: Health-Society Interaction', Department of Public Health Science, Graduate School, Korea University, Seoul 02841, Korea; honghyok@korea.ac.kr (Ho.K.); ippiy@hanmail.net (Hy.K.); garam0110@gmail.com (G.B.); 91.minhle@gmail.com (M.L.); yschoi860@gmail.com (Y.C.)
[4] School of Health Policy and Management, College of Health Science, Korea University, Seoul 02841, Korea
* Correspondence: jtlee@korea.ac.kr; Tel.: +82-2-3290-5668

Int. J. Environ. Res. Public Health **2019**, *16*, 173; doi:10.3390/ijerph16020173

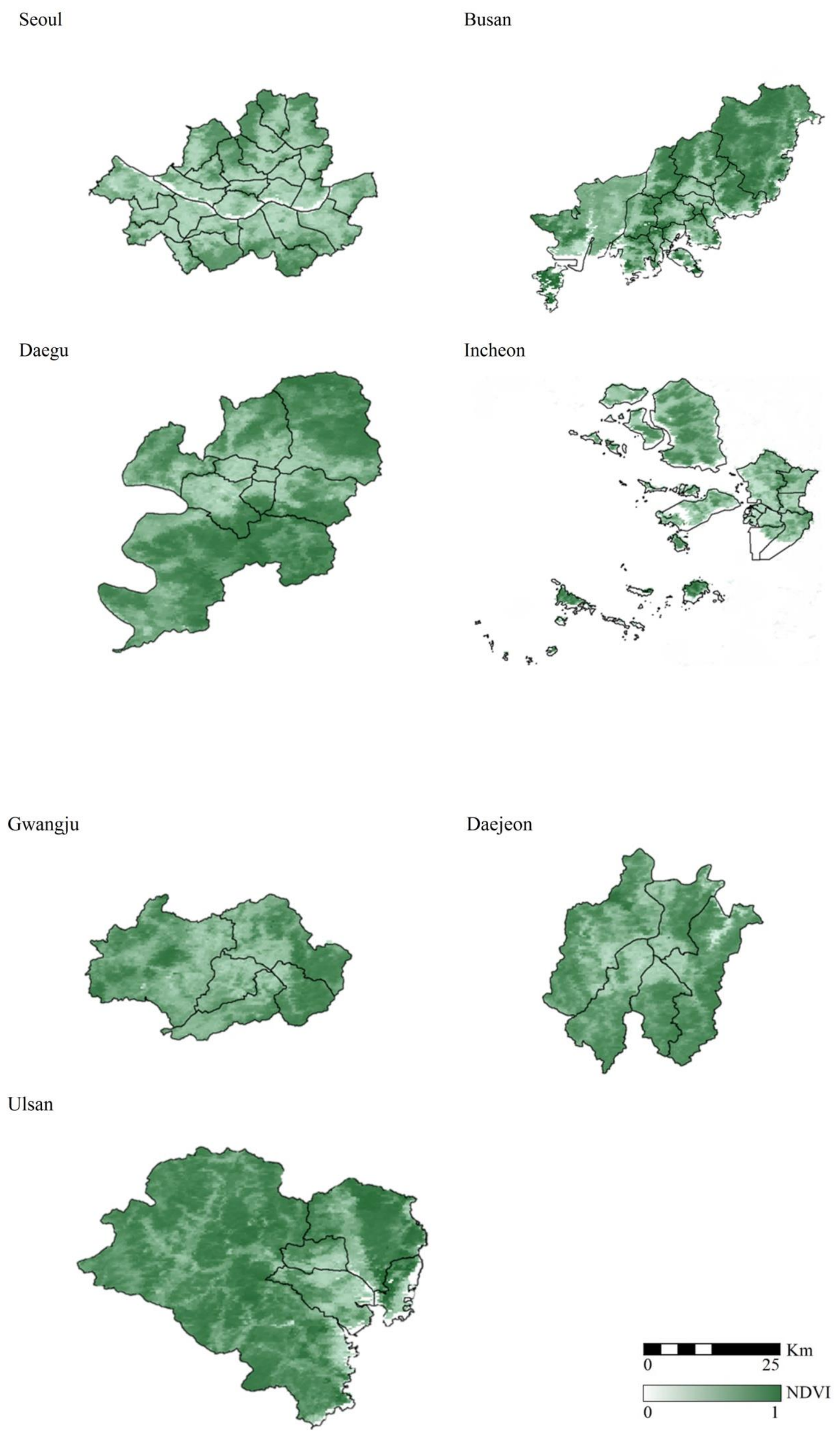

Figure S1. NDVI image of the seven major cities in Korea, 16 October 2009.

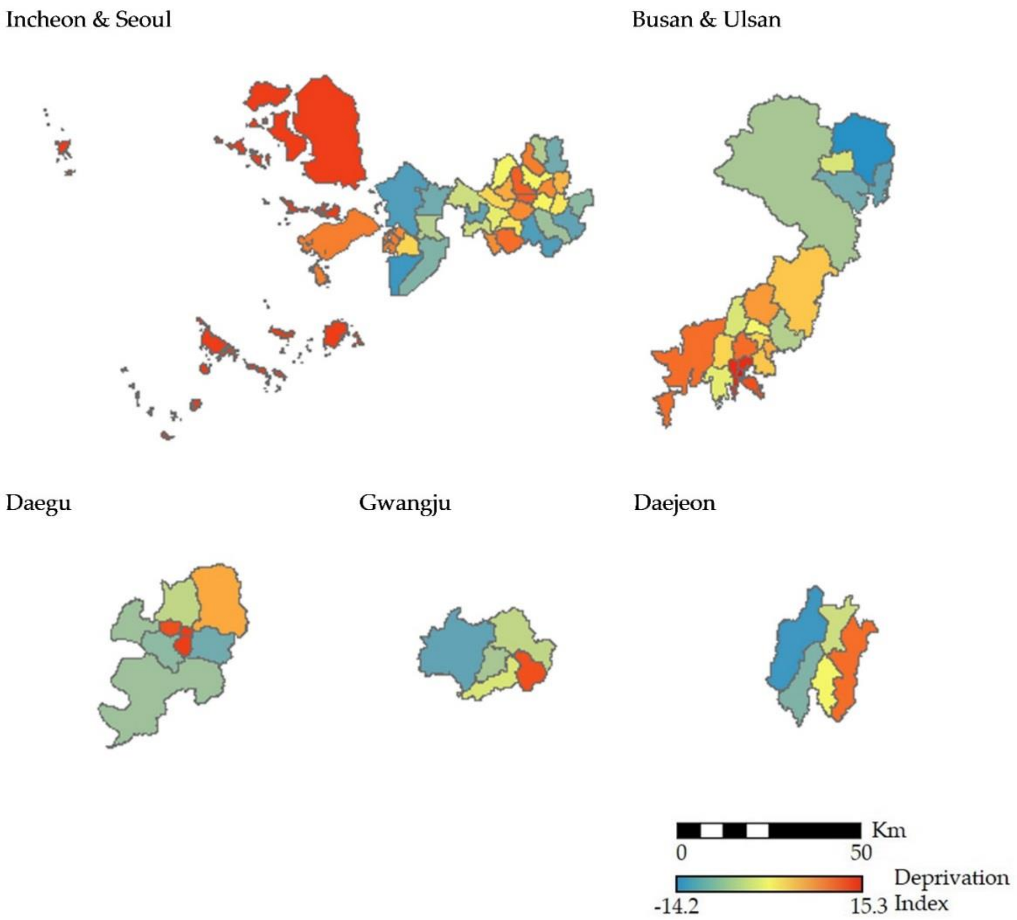

Figure S2. Deprivation index of the 74 districts in the seven major cities in Korea. A higher deprivation index value indicates more deprived.

Table S1. Indicators for greenness and their correlations for the 74 districts in the seven major cities in Korea.

	**NDVI**	**FA** [a] **(ha)**	**FV** [b] **(m^3)**	**FA per district area (%)**	**FV per district area (m^3/ha)**	**FA per capita (ha/person)**	**FV per capita (m^3/person)**
NDVI		0.57 *	0.61 *	0.94 *	0.94 *	0.38 **	0.43 **
FA (ha)			0.99 *	0.52 *	0.44 *	0.55 *	0.62 *
FV (m^3)				0.54 *	0.50 *	0.49 *	0.57 *
FA per district area (%)					0.93 *	0.37 **	0.41 **
FV per district area (m^3/ha)						0.23 **	0.29 **
FA per capita (ha/person)							0.99 *
FV per capita (m^3/person)							

[a] Forest area; [b] Forest volume. * p-value < 0.001; ** p-value < 0.05.
